# LSPR Tunable Ag@PDMS SERS Substrate for High Sensitivity and Uniformity Detection of Dye Molecules

**DOI:** 10.3390/nano12213894

**Published:** 2022-11-04

**Authors:** Xiaoya Yan, Hongyan Shi, Pengxue Jia, Xiudong Sun

**Affiliations:** 1 School of Physics, Harbin Institute of Technology, Harbin 150001, China; 2 Key Laboratory of Micro-Nano Optoelectronic Information System of Ministry of Industry and Information Technology, Harbin 150001, China; 3 Key Laboratory of Micro-Optics and Photonic Technology of Heilongjiang Province, Harbin Institute of Technology, Harbin 150001, China; 4 Collaborative Innovation Center of Extreme Optics, Shanxi University, Taiyuan 030006, China

**Keywords:** sensitivity, uniformity, bio-template-stripping, FDTD simulation, qualitative detection

## Abstract

At present, the use of efficient and cost-effective methods to construct plasmonic surface-enhanced Raman scattering (SERS) substrates of high sensitivity, uniformity and reproducibility is still crucial to satisfy the practical application of SERS technology. In this paper, a localized surface plasmonic resonance (LSPR) tunable flexible Ag@PDMS substrate was successfully constructed by the low-cost bio-template-stripping method and magnetron sputtering technology. The theory proves that the local electromagnetic field enhancement and “hot spot” distribution is adjustable by modifying the size of the optical cavity unit in the periodicity nanocavity array structure. Experimentally, using rhodamine 6G (R6G) as the target analyte, the SERS performance of optimal Ag@PDMS substrate (Ag film thickness for 315 nm) was researched in detail, which the minimum detection limit was 10^−11^ M and the enhancement factor was calculated as 8.03 × 10^8^, indicating its high sensitivity. The relative standard deviation (RSD) was calculated as 10.38%, showing that the prepared substrate had excellent electromagnetic field enhancement uniformity. At last, the trace detection of Crystal violet (CV, LOD = 10^−9^ M) and the simultaneous detection of three common dyes (R6G, CV and Methylene blue (MB) mixture) were also realized. This result suggests that the SERS substrate has a good application prospect in the quantitative and qualitative detection of dye molecules.

## 1. Introduction

SERS as a trace detection technology can provide unique spectra vibration information of Raman molecules, and it is used to identify and quantify ultra-low levels analyte in rapid, robust and nondestructive detection conditions [1]. The technology is widely applied in pesticide residue [2], cancer diagnosis [3], biological analysis [4,5], environmental monitoring [6] and other fields. The overall SERS enhancement is contributed by chemical enhancement (CM) and electromagnetic enhancement (EM) [7]. In particular, the strong electromagnetic field enhancements are closely related to “hot spots” and are excited at the metal nanostructure gap with less than 10 nm [8]. When the probe molecule enters the “hot spots”, the Raman signal can be greatly amplified and even to the single-molecule detection level due to the LSPR effect [9]. Therefore, the construct of highly sensitive and uniform plasmonic substrates is crucial to satisfy the practical application of SERS technology.

At present, utilizing precious metals (such as Au, Ag and Cu) to synthesize colloid nanoparticles and designing rigid nanostructures are two commonly used strategies of prepare plasmonic substrates [2]. Thereinto, the synthesis of colloid nanoparticles is cost-efficient and easy to control. However, it is difficult to assure the uniformity and repeatability of SERS spectra by using the colloid system as a plasmonic substrate [10,11]. On the contrary, the rigid nanostructures fabricated via various lithography technologies ensure the uniformity and repeatability of SERS signals on a large area, but the extensive applications in practical detection have been restricted due to the expensive, time-consuming and cumbersome preparation procedures [12,13]. Therefore, it is still necessary to explore the preparation of plasmonic substrates that can simultaneously take into account the cost-effectiveness, preparation procedure, and SERS performance. For example, Aybeke et al. prepared high-performance SERS-active substrates using inexpensive, fast and easy thermal synthesis and conventional physical vapor deposition methods [14,15]. Lately, the flexible SERS-active substrates were prepared via modifying precious metal nanoparticles on the flexible support materials, such as polydimethylsiloxane (PDMS) [16] and polymethylmethacrylate (PMMA) [17], which attracted widespread attention due to its simple operation and low-cost.

Although flexible SERS-active substrates possess many inherent advantages, the local electromagnetic field enhancement of SERS substrates is still strongly dependent on their geometric structure [18,19]. Among them, some engineering “hot spots” are excited on the ordered micro-nano structure arrays, not only having sensitive SERS signals but also “hot spots” distributed uniformly [5,20,21,22]. For example, Kozhina et al. used a cost-effective template-assisted electrodeposition method to grow metal nanowires (NWs) in polymer trace etching film (TM) holes to prepare SERS substrates [21,22]. Xiang et al. obtained an Au/AAO SERS substrate by sputtering a gold nano-film onto the porous anodic aluminum oxide (AAO) surface [23]. According to the effective medium theory (EMT), the porous structure had excellent SERS performance because the effective refractive index gradient was formed when the incident light from air irradiated to the substrate surface, which leads to the light propagation path being prolonged and ultimately realized the light-trapping effect [24]. Unfortunately, the preparation process of AAO has high requirements for experimental conditions and operators. The natural biological systems after a long period of evolution have developed many exquisite micro/nano structures, such as lotus leaves, butterfly wings, and cicada wings (CWs) [25,26,27]. In particular, on the transparent CWs surface presence of large-area hexagonal tapered nanopillars arrays structures [28]. Bio-template-stripping as a cost-effective and no-clean approach is widely used to transfer nanostructures [29]. For instance, Liu and Wang et al. used the bio-template-stripping method to transfer the nanopillars arrays structure on the cicada wings surface and to study the superhydrophobic and anti-reflective properties of the transferred structure [30,31]. Nevertheless, the SERS effect based on this structure has rarely been reported.

According to the above facts, the CWs with large-area hexagonal tapered nanopillars arrays structures were used as the original template, and plasmonic Ag@PDMS SERS substrates of high sensitivity, uniformity and reproducibility were designed through the bio-template-stripping method and magnetron sputtering technology. Theoretically, the local electromagnetic field enhancement and “hot spot” distribution based on the resonance coupling effect was studied by modifying the size of the optical cavity unit in the periodicity nanocavity array structure. Experimentally, the sensitivity, uniformity and reproducibility of the optimal Ag@PDMS substrates were researched in detail. At last, the trace detection of CV and the simultaneous detection of three common dyes (the mixture of R6G, CV and MB) were also studied. The research results suggest that this substrate could be a potential candidate in the quantitative and qualitative detection of dye molecules.

## 2. Materials and Methods

### 2.1. Materials and Instruments

CWs were purchased from Beijing Jiaying Grand Life Sciences Co., Ltd. (Beijing, China); PDMS and curing agent were purchased from Dow Corning Co., Ltd. (Shenzhen, China); Acetone and ethanol were obtained from Sinopharm Chemical Reagent Corporation (Shanghai, China); R6G, CV, and MB were purchased from Tianjin Zhiyuan Chemical Reagent Co., Ltd. (Tianjin, China); Ultra-pure water was prepared with a Milli-Q water purification system.

The surface morphology of the substrate was characterized through Field Emission Scanning Electron Microscopy (FE-SEM, Zeiss Merlin Compact, Oberkochen, Germany). Ag films were sputtered by a direct-current (DC) magnetron sputtering instrument (MSD800, Beijing, China). The thickness of Ag films was measured by Probe Profiler (KLA/Tencor D-100, Silicon Valley, CA, USA). The reflectance spectra were measured by PerkinElmer Lamda (950 UV/Vis/NIR, Waltham, MA, USA). The SERS spectra (NTEGRA Spectra, Moscow, Russia) were obtained on a Raman spectrometer using a 532 nm laser source (MS 5004i, Graben, Germany).

### 2.2. Fabrication of Ag@PDMS Substrate

**Figure 1:**
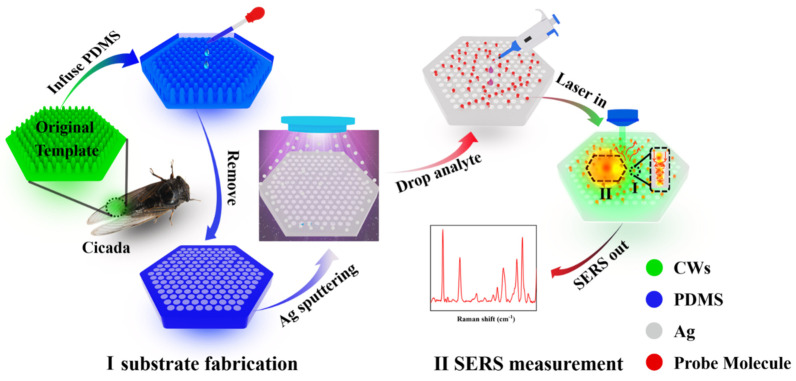
The preparation process of Ag@PDMS substrate and its SERS measurement.

As shown in Figure 1, the fabrication procedure of the Ag@PDMS SERS-active substrate was displayed. The detailed steps were as follows: 

(a) Pretreatment of original template CW. First, the CW was cut to eliminate the support network of the edge part and obtain a size of 1 × 1.5 cm^2^ sample. Then, the wing was cleaned in acetone, ethanol and ultra-pure water for 10 min, respectively, to remove the surface adhesive stain. After that, the clean biological specimen was dried naturally and stored carefully for later use. 

(b) Preparation of flexible PDMS film. First, PDMS prepolymer and curing agent were mixed evenly in a weight ratio of 10:1, and the mixture was let stand for 30 min to exclude the small bubbles. Next, the mixture was dropped onto the pretreated CW and followed by a heat solidification under an 80 °C incubator for 2 h. After that, taking the original template CW off carefully, a transparent thin film with large-area nanocavities structure was manufactured successfully.

(c) Deposition of Ag film. DC magnetron sputtering instrument was used to decorate different thickness Ag films on the above-prepared PDMS film surface. The current and voltage in the sputtering process were fixed at 0.13 A and 0.35 KV, respectively. The sputtering rates were approximately 35 nm/min. The prepared Ag@PDMS SERS-active substrate was stored in a vacuum seal bag to mitigate surface oxidation and environmental disturbances.

### 2.3. SERS Measurements and Enhancement Factor Calculation

The standard solutions (10^−3^ M) of R6G, CV and MB were all prepared by dissolving the solid powder in water. Then the standard solutions were step-to-step diluted to different concentrations (from 10^−3^ M to 10^−11^ M for R6G, from 10^−3^ M to 10^−9^ M for CV and from 10^−3^ M to 10^−6^ M for MB). For evaluating the SERS activity and screening out the optimal substrate, 10^−6^ M R6G solutions were dripped onto the different structure Ag@PDMS substrates, and the SERS signals were obtained after drying naturally. To investigate the SERS sensitively for different dye molecules, a series of concentrations of R6G and CV analytes were dripped on the optimal Ag@PDMS substrate for SERS measurements. To study the qualitative detection capability of the optimal substrate, the 10^−6^ M R6G, CV and MB with the same volume were mixed, and the mixture was dripped onto the substrate surface and dried naturally before SERS measurements. All of the Raman spectra were collected using a 532 nm laser as the excitation source, and the acquisition time was 3 s. The size of the sample used in the SERS measurement process was 0.5 × 0.5 cm^2^. For a more vivid description, the process of SERS measurements was shown in Figure 1.

In addition, to further estimate the enhancement performance of optimal Ag@PDMS substrate, the SERS signals of R6G onto the PDMS film (10^−3^ M) and the prepared Ag@PDMS substrate (10^−10^ M) were used as a reference. The following formula was employed for EF calculations [32]:

(1) $$\mathrm{EF}=\frac{I_{\mathrm{SERS}}\times N_{\mathrm{RS}}}{I_{\mathrm{RS}}\times N_{\mathrm{SERS}}}$$

Herein, *I*_SERS_ and *I*_RS_ represent the peak integral intensity of the SERS spectrum and Raman spectrum, respectively. NSERS and NRS represent the concentration of R6G solutions in the SERS measurement and Raman measurement, respectively.

### 2.4. Theoretical Simulations of an Optical Cavity Unit Based on Ag@PDMS Substrate

The SERS performance of the substrate is closely related to the local electromagnetic field enhancement and “hot spot” distribution [33,34]. In particular, in the periodicity nanocavity array structure, the enhancement performance is mainly determined by the cavity size. Therefore, to further analyze the absorption, electric field enhancement and “hot spot” distribution of different optical cavity size periodicity nanocavity array structures based on Ag@PDMS structures, the Finite-difference time-domain (FDTD) simulations were carried out. First of all, the nanorod array structure from the CW surface was used as the construction basis to obtain the initial periodicity nanocavity array structure. The cavity depth, top radius and bottom radius of the initial single optical cavity unit are 270, 72 and 34 nm, respectively. Then, the absorption and electric field enhancement of periodicity nanocavity array structures with single cavity sizes ranging from hundreds to tens of nanometers were studied.

The optical cavity unit and hexagonal nanocavity array structures were covered with vacuum placed on SiO_2_ substrate. Both vacuum and the SiO_2_ substrate were semi-infinite. The boundary conditions in the optical cavity unit simulation were set as perfectly matched layer (PML). In the hexagonal nanocavity array structure simulation, the *X* and *Y* axis directions were set to periodic boundary conditions, and the *Z* axis direction was PML. The material properties of Ag were gained from the software material library. The total-field scattered-field light source with a wavelength ranging from 300 to 900 nm was used. The frequency-domain field and power monitor were applied to record the changes of the electric-field intensity and distribution. The mesh step size is 3 nm in both the optical cavity element and the hexagonal nanocavity array structure. The sizes of the simulation regions in the optical cavity unit and hexagonal nanocavity array structures are 1 × 1 × 1 µm^3^ and 0.55 × 0.55 × 1 µm^3^, respectively. In the optical cavity unit, the distance from the structure to the top and surrounding PML boundaries is 0.35 µm and 0.426 µm, respectively.

## 3. Results and Discussion

### 3.1. Characterization and Optimization of the Ag@PDMS Substrate

**Figure 2:**
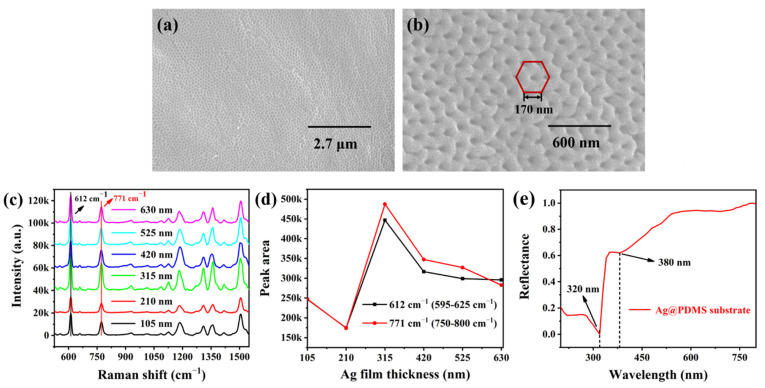
Ag@PDMS substrate characterization and optimization. SEM images of Ag@PDMS substrate with Ag film thickness of 210 nm at the (**a**) micro and (**b**) nano levels. (**c**) The SERS spectra of 10^−6^ M R6G adsorbed on different Ag@PDMS substrates, and (**d**) the variation trend of integrated intensities at 612 cm^−1^ (595−625 cm^−1^) and 771 cm^−1^ (750−800 cm^−1^) peaks. (**e**) Reflectance spectra of Ag_(315)_@PDMS substrate.

The micro-level SEM characterization of the Ag@PDMS substrate with an Ag film thickness of 210 nm was displayed in Figure 2a. It can be discovered intuitively that the large-area and uniformly porous array structure has successfully been fabricated through the bio-template-stripping method. An enlarged SEM image of Figure 2a was presented in Figure 2b, and the center distance between adjacent structures is about 170 nm. 

To investigate the local electromagnetic field enhancement performance, 10^−6^ M R6G is selected to test the SERS performance of the different Ag@PDMS substrates, as shown in Figure 2c. As seen from the SERS spectra, the main characteristic peaks of SERS substrates for decorating different thickness Ag films are in the same position. The peaks at 612, 771 and 1183 cm^−1^ are C-C-C bond stretching vibration, C-H out-plane bending vibration and C-H in-plane bending, respectively. The peaks at 1363 and 1511 cm^−1^ are generally associated with aromatic C-C tensile vibration [35,36]. The peak intensity reached a maximum value when the Ag film thickness for 315 nm. In addition, with the increase of Ag film thickness, the variation trend of integrated intensities at 612 cm^−1^ (595–625 cm^−1^) and 771 cm^−1^ (750–800 cm^−1^) peaks were illustrated in Figure 2d. The formation of such a trend lies in the fact that many high-effective “hot spots” could be caused in a porous array structure of Ag film thickness for 315 nm, inducing much significant SERS signal under excitation [37,38,39]. Based on the above result, the Ag@PDMS structure (named “Ag_(315)_@PDMS”) with an Ag film thickness of 315 nm is selected to evaluate the SERS performance in the following investigation. To prove the plasmonic properties of the prepared Ag_(315)_@PDMS substrate, a reflectance spectrum in the range from 200 to 800 nm was measured, as shown in Figure 2e. Due to the presence of 315 nm Ag film, the transmittance in the substrate is eliminated, so the absorbance is strong at low reflectance in this system. Two vertical black dotted lines are located at 320 and 380 nm in Figure 2e, indicating the positions of the plasmon resonance peaks. The strong resonance peak at 320 nm originates from the cavity mode [40]. The weak resonance peak at 380 nm corresponds to the surface plasmon polariton (defined as the SPP mode), which propagates along the top of the nanocavity film surface [41].

### 3.2. Theoretical Simulations of an Optical Cavity Unit Based on Ag@PDMS Substrate

**Figure 3:**
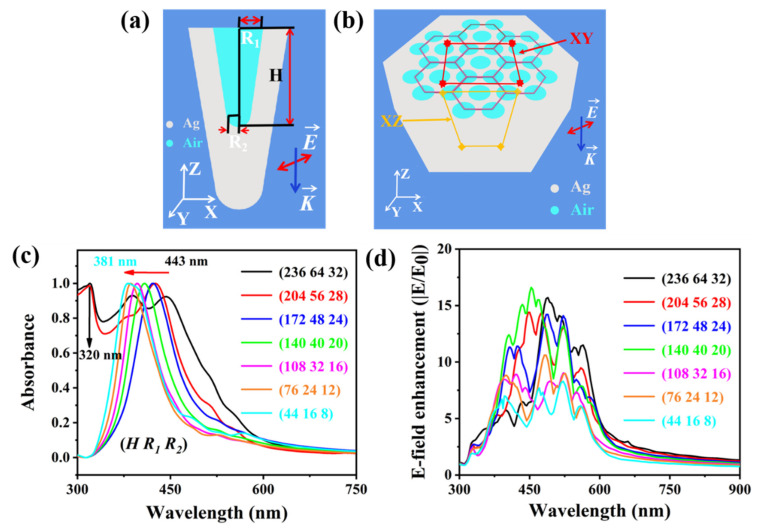
(**a**,**b**) The model diagram of an optical cavity unit and periodicity nanocavity array structure in Ag@PDMS nanostructure. (**c**) The absorption spectra of the periodic nanocavity array structure composed of conical cavities size in the range of hundreds to tens of nanometers. (**d**) The dependence of the maximum electric field enhancement on the incident light wavelength.

The model diagram of an optical cavity unit in Ag@PDMS nanostructure was constructed in Figure 3a. Thereinto, the H (236~44 nm), R_1_ (64~16 nm) and R_2_ (32~8 nm) show the cavity depth, top radius and bottom radius of the optical cavity unit, respectively, and the nanocavities with different sizes were named as (H, R_1_, R_2_). The red arrow $\vec{E}$ and blue arrow $\vec{K}$ indicates the polarization direction and propagation direction of the incident light. To reflect the true nature of the fabricated substrate and study the influence of the periodicity of the nanocavity and the continuity of the metal film for the local electromagnetic field, a hexagonal array structure composed of conical cavities was constructed. The model diagram of a periodicity nanocavity array structure in Ag@PDMS substrate was constructed in Figure 3b.

The LSPR peculiarity is strongly dependent on the geometry and size of the plasmonic nanostructure. Therefore, under the depth-to-diameter ratio unchanging condition, the absorption spectra of the periodic nanocavity array structure composed of conical cavities in the range of hundreds to tens of nanometers size were shown in Figure 3c. It can be noticed that the resonance peaks based on the coupling effect are highly modulated with the size of the conical cavity. In particular, when the conical cavity depth is less than 200 nm, the resonance peak at 320 nm originates from the cavity mode was disappear, which is consistent with the previously reported results [40]. As the size of the nanocavity decreases, the SPP mode blue shifts, which may be due to the gradual dominance of the SPP mode in this process (443–381 nm), leading to the weakening of the coupling effect between the cavity mode [42]. By comparing Figure 2e and Figure 3c, it can be found that there are some differences between the plasmon resonance peaks. Compared with theoretical simulations, the cavity mode observed in the experimental measurements may be due to the uniform film-forming method of silver nanoparticles over a large area so that the nanocavity depth always is greater than 200 nm, while the weak SPP mode may be caused by the roughness of the top surface of the substrate. It is known that the sensitivity detection based on the SERS substrate is closely related to the near-field electromagnetic field enhancement of the plasmonic nanostructure [37]. Therefore, the dependence of the maximum electric field enhancement on the incident light wavelength is also researched, as shown in Figure 3d, where E_0_ and E represent the incident electric field intensity and the local electric field intensity, respectively. It can be observed that all the maximum electric field enhancement is less than 20, due to the absence of high-efficient “hot spots”.

**Figure 4:**
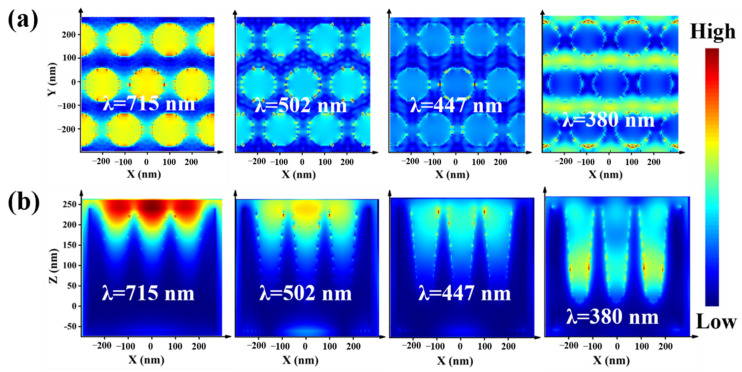
The “hot spot” distribution of the hexagonal nanocavities array structure in the (**a**) XY and (**b**) XZ plane under the 715, 502, 447 and 380 nm wavelength.

The “hot spot” distribution of a hexagonal nanocavity array unit at different wavelengths was researched, as shown in Figure 4, and the size of each conical cavity unit was (236, 64, 32) nm. The electromagnetic field distribution of the hexagonal nanocavities array structure under the XY and XZ plane was studied at 715, 502, 447 and 380 nm wavelength, as shown in Figure 4a,b. It can not only be it found that the “hot spot” in the optical cavity unit gradually moves downward with the decreasing of the wavelength, but also be found that the strong electromagnetic coupling formed between the nanocavities.

### 3.3. SERS Performances of the Ag@PDMS Substrate

**Figure 5:**
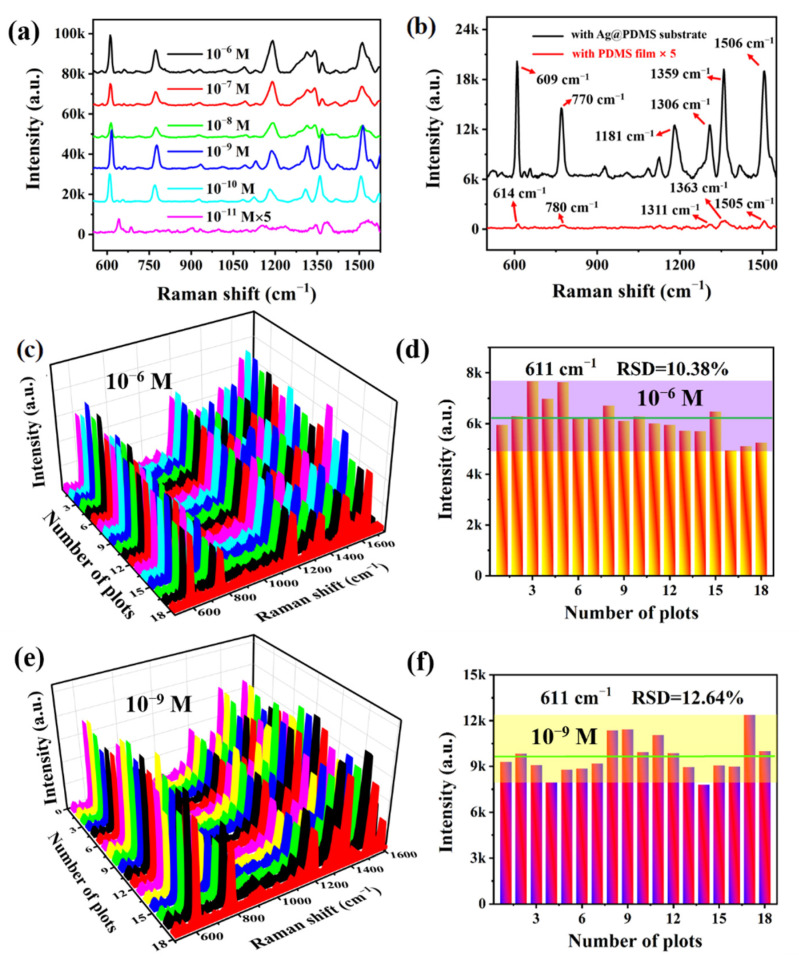
SERS performances of Ag_(315)_@PDMS substrate. (**a**) SERS spectra of different concentrations R6G adsorbed on the Ag_(315)_@PDMS substrate. (**b**) The SERS spectrum of 10^−10^ M R6G adsorbed on the Ag_(315)_@PDMS substrate and Raman spectrum of 10^−3^ M R6G adsorbed on the PDMS film. (**c**,**e**) SERS spectra of 10^−6^ and 10^−9^ M R6G solution obtained from 18 randomly selected positions from 3 Ag_(315)_@PDMS substrates. (**d**,**f**) The signal intensity distribution at the Raman characteristic peak of 611 cm^−1^ in Figure 6c,e.

The sensitivity is one of the important parameters in the trace detection. To research the sensitivity of Ag_(315)_@PDMS substrate, the Raman signals of different concentrations of R6G (10^−11^–10^−6^ M) were detected, as depicted in Figure 5a. It can be observed that the typical Raman peaks were still visible even at a low concentration of 10^−11^ M, indicating that the Ag_(315)_@PDMS substrate possesses excellent sensitivity. To more intuitively evaluate the electromagnetic field enhancement capability of the optimal substrate, the SERS spectrum of 5 μL 10^−10^ M R6G solution on the Ag_(315)_@PDMS substrate and the Raman spectrum of 5 μL 10^−3^ M R6G solution on the PDMS film were displayed in Figure 5b. The integral intensity at peak 609 cm^−1^ is used for calculation, whose value is 169,460.75 for R6G (10^−10^ M) on the optimal Ag_(315)_@PDMS substrate, while is 2110.13 for R6G (10^−3^ M) on the PDMS film. Therefore, we could calculate *I*_SERS_/*I*_RS_ = 80.3 and *N*_RS_/*N*_SERS_ = 10^7^, put into Formula (1), the EF was finally calculated for 8.03 × 10^8^. The high enhancement capability can be attributed to the nanostructure resonance coupling effect and the superposition effect between array structures.

The reproducibility and uniformity of the SERS-active substrate are critical in practical applications. To verify the batch-to-batch signal reproducibility, 10^−6^ and 10^−9^ M R6G solution was adsorbed on 3 batches of Ag_(315)_@PDMS substrate, and randomly selected at 18 points to collect the SERS spectra, as shown in Figure 5c,e. The almost identical spectra curves and peak intensities indicate that the Ag_(315)_@PDMS substrate has wonderful reproducibility. Figure 5d,f exhibits the peak intensities of the Raman characteristic peak of 611 cm^−1^ in Figure 5c,e, in which the green horizontal line and the rectangular area represents the average intensity and the intensity deviation range, respectively. The RSD of the 10^−6^ and 10^−9^ M R6G Raman characteristic peak at 611 cm^−1^ is 10.38% and 12.64%, further revealing that the Ag_(315)_@PDMS substrate has excellent reproducibility [43].

**Figure 6:**
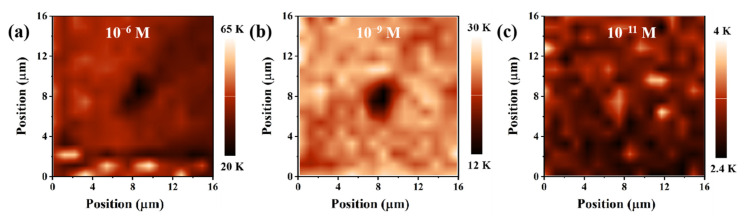
The integral intensity mapping image of (**a**) 10^−6^, (**b**) 10^−9^ and (**c**) 10^−11^ M R6G solution at peak 612 cm^−1^ (595–625 cm^−1^).

The SERS mapping of images of Ag_(315)_@PDMS substrate are accomplished to evaluate the spot-to-spot uniformity. The integral intensity mapping of 10^−6^ and 10^−9^ M R6G solution at the peak of 612 cm^−1^ (595–625 cm^−1^) was plotted in Figure 6a,b. The SERS mapping was acquired on a 16 μm × 16 μm square area region, and the step length was set to 1 μm. Each pixel of the mapping images indicated the integral intensity of R6G at a spatial position on the substrate, and the relatively uniform color distribution indicates that the SERS substrate has excellent spot-to-spot uniformity. Some very low integral intensity in SERS mapping images appeared due to the substrate surface being contaminated. Meanwhile, to explore the dependability of trace detection at low concentration conditions, the integral intensity mapping image of 10^−11^ M R6G solution at the peak of 612 cm^−1^ (595–625 cm^−1^) was also plotted, as shown in Figure 6c. The sporadic SERS signal was still observed even though the 10^−11^ M R6G can’t form a monolayer film on the Ag@PDMS substrate surface.

### 3.4. Practical Application of the Ag@PDMS SERS Substrate

**Figure 7:**
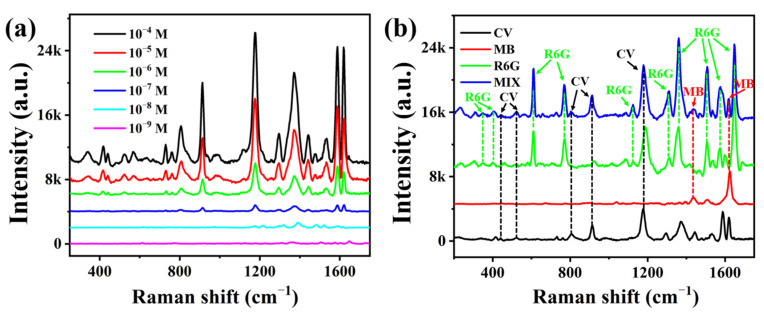
Quantitative and qualitative detection of dye molecules by Ag_(315)_@PDMS substrate. (**a**) SERS spectra of CV with different concentrations adsorbed on the Ag_(315)_@PDMS substrate. (**b**) SERS spectra of CV (black line), MB (red line), R6G (green line) and their mixtures (blue line).

As a widely used in the textile, pharmaceutical and aquaculture industries mitotic poisoning agent dye, CV is utilized as a target pollutant to evaluate the practical application potential of the Ag_(315)_@PDMS substrate [44,45]. As presented in Figure 7a, the SERS spectra of CV with different concentrations were exhibited. It can be found that the location of vibration peaks was consistent with the reported in the literature previously [20], and the minimum detection line was 10^−9^ M. The detection limit discrepancy between R6G and CV may be due to the different in intrinsic properties and the affinities to metal surfaces [46]. In addition, the SERS spectrum has been widely used in the qualitative detection of several substances due to narrow-band molecularly peaks. As R6G, CV and MB are three common illegal ingredients in food, identifying these substances effectively from the mixture is important. Based on this, the SERS spectra of R6G, CV, MB and their mixture solution were compared in Figure 7b. The Raman characteristic peaks of the three dyes are identified clearly from the SERS spectrum of mixture solution, which shows that the Ag@PDMS substrate as a fascinating SERS platform can simultaneous detection several substances in practical application.

## 4. Conclusions

To sum up, by sputtering different thickness Ag films on the ordered flexible PDMS nanostructure surface, LSPR tunable Ag@PDMS SERS substrate was successfully constructed. Using R6G as the target analyte, the SERS detection results indicated that the Ag@PDMS substrate had the optimal local electromagnetic field enhancement at the Ag film thickness for 315 nm. The SERS performance of the optimal substrate was also researched in detail, whose minimum detection limit (LOD = 10^−11^ M) and EF (8.03 × 10^8^) indicated its high sensitivity. The mapping images and RSD (10.38%) showed that the substrate had excellent electromagnetic field enhancement uniformity and reproducibility. In addition, the local electromagnetic field enhancement and “hot spot” distribution were calculated through numerical simulation. The results indicate that the resonance coupling effect is adjustable by modifying the size of the optical cavity unit in the periodicity nanocavity array structure. At last, the trace detection of CV (LOD = 10^−9^ M) and the simultaneous detection of three common dyes (R6G, CV and MB mixture) were also realized. The results suggest that this substrate could as a potential candidate in the quantitative and qualitative detection of dye molecules.

## Data Availability

The data are available upon reasonable request from the corresponding author.
